# A robust data scaling algorithm to improve classification accuracies in biomedical data

**DOI:** 10.1186/s12859-016-1236-x

**Published:** 2016-09-09

**Authors:** Xi Hang Cao, Ivan Stojkovic, Zoran Obradovic

**Affiliations:** 1 Center for Data Analytics and Biomedical Informatics, College of Science and Technology, Temple University, 1925 North 12th Street, Philadelphia, 19122 USA; 2 Signals and Systems Department, School of Electrical Engineering, Bulevar kralja Aleksandra 73, Belgrade, 11120 Serbia

**Keywords:** Data scaling, Data normalization, Outlier, Classification model, Generalized logistic function, Empirical cumulative distribution function

## Abstract

**Background:**

Machine learning models have been adapted in biomedical research and practice for knowledge discovery and decision support. While mainstream biomedical informatics research focuses on developing more accurate models, the importance of data preprocessing draws less attention. We propose the Generalized Logistic (GL) algorithm that scales data uniformly to an appropriate interval by learning a generalized logistic function to fit the empirical cumulative distribution function of the data. The GL algorithm is simple yet effective; it is intrinsically robust to outliers, so it is particularly suitable for diagnostic/classification models in clinical/medical applications where the number of samples is usually small; it scales the data in a nonlinear fashion, which leads to potential improvement in accuracy.

**Results:**

To evaluate the effectiveness of the proposed algorithm, we conducted experiments on 16 binary classification tasks with different variable types and cover a wide range of applications. The resultant performance in terms of area under the receiver operation characteristic curve (AUROC) and percentage of correct classification showed that models learned using data scaled by the GL algorithm outperform the ones using data scaled by the Min-max and the Z-score algorithm, which are the most commonly used data scaling algorithms.

**Conclusion:**

The proposed GL algorithm is simple and effective. It is robust to outliers, so no additional denoising or outlier detection step is needed in data preprocessing. Empirical results also show models learned from data scaled by the GL algorithm have higher accuracy compared to the commonly used data scaling algorithms.

## Background

There is an increasing interest in research and development of machine learning and data mining techniques for aid in biomedical studies as well as in clinical decision making [1–4]. Typically, statistical learning methods are performed on the data of observed cases to yield diagnostic or prognostic models that can be applied in future cases in order to infer the diagnosis or predict the outcome. Such learned models might be used to assist physicians in guiding their decisions, and are sometimes shown to outperform the experts’ prediction accuracy [5]. Furthermore, such models can discover previously unrecognized relations between the variables and outcome improving knowledge and understanding of the condition. Such discoveries may result in improved treatments or preventive strategies. Given that predictive models compute predictions based on information of a particular patient, they are also promising tools for achieving the goal of personalized medicine.

Predictive models have huge potential because of their ability to generalize from data. Even though predictive models lack the skills of a human expert, they can handle much larger amounts of data and can potentially find subtle patterns in the data that a human could not. Predictive models rely heavily on training data, and are dependent on data quality. Ideally, a model should extract the existing signal from the data and disregard any spurious patterns (noise). Unfortunately, this is not an easy task, since data are often far from perfect; some of the imperfections include irrelevant variables, small numbers of samples, missing values, and outliers.

Therefore, data preprocessing is common and necessary in order to increase the ability of the predictive models to extract useful information. There are various approaches targeting different aspects of data imperfection; such as imputations for missing values, smoothing for removing the superimposed noise, or excluding the outlier examples. Then there are various transformations of variables, from common scaling and centering of the data values, to more advanced feature engineering techniques. Each of those techniques can make a significant improvement in predictive model performance when learned on the transformed data.

### Data scaling in classification modeling

In the machine learning and data mining community, data scaling and data normalization refer to the same data preprocessing procedure, and these two terminologies are used interchangeably; their aim is to consolidate or transfer the data into ranges and forms that are appropriate for modeling and mining [6]. Models trained on scaled data usually have significantly higher performance compared to the models trained on unscaled data, so data scaling is regarded as an essential step in data preprocessing. Data scaling is particularly important for methods that utilize distance measures, such as nearest neighbor classification and clustering. In addition, artificial Neural Network models require the input data to be normalized, so that the learning process can be more stable and faster [7].

**Confusions of gene expression normalization** In medicine, gene expression data obtained from microarray technology are widely used for disease/cancer diagnosises. Usually, a normalization step is conducted for the purpose of identifying and removing sources of systematic variation in the measured fluorescence [8], before the data are ready for analysis. However, the gene expression normalization step is not equivalent to the data scaling step that we study in this context. In most cases, a normalized gene expression dataset needs to be processed/scaled by a data scaling step before learning a classification model. The models that are learned from gene expression data with scaling usually outperform the models that are learned from gene expression data without scaling, with considerable margins.

### Commonly used data scaling algorithms

Two data scaling algorithms are widely used: Min-max algorithm and Z-score algorithm.

**Min-max algorithm** In the Min-max algorithm, the original data are linearly transformed. We denote *x*_*min*_ and *x*_*max*_ as the minimum and the maximum of a variable in the samples. The Min-max algorithm maps a value, *v*, of this variable to a value, *v*^′^, using the following formula:

1 $$\begin{array}{*{20}l} v' = \frac{v-x_{min}}{x_{max}-x_{min}} + x_{min}. \end{array} $$

The Min-max algorithm scales a variable in the training samples in the interval of [ *x*_*min*_, *x*_*max*_] to [-1, 1] (or [0, 1]) by using a linear mapping. However, when the unseen/testing samples fall outside of the training data range of the variable, the scaled values will be out of the bounds of the interval [-1, 1] (or [0, 1]), and that may pose problems in some applications; in addition, it is very sensitive to outliers, as shown in latter sections.

**Z-score algorithm** In the Z-score algorithm, the new value, *v*^′^, of a variable, is scaled from the original value, *v*, using the formula:

2 $$\begin{array}{*{20}l} v' = \frac{v-\bar{x}}{\sigma_{x}}, \end{array} $$

where $\bar {x}$ and *σ*_*x*_ are the mean and standard deviation of the variable values in the training samples, respectively. After the scaling, the new values will have value 0 as the mean, and value 1 as the standard deviation. This algorithm does not map the original data into an interval, and it is also sensitive to outliers. When the number of examples is small, especially in scenarios in biomedical research, the mean and standard deviation calculated from the data may not be able to approximate the true mean and standard deviation well, so future input values will be scaled poorly.

## Methods

The idea of the GL algorithm for data scaling is adapted from the histogram equalization technique, and it can map both the original and future data into a desired interval. The algorithm has no assumption on the sample distribution and utilizes generalized logistic functions to approximate cumulative density functions. Since it maps data into a uniformly distributed range of values, the points that were previously densely concentrated on some interval become more discernible, which allows more room for representation of the subtle differences between them. In addition, the GL algorithm reduces the distance of outliers from other samples, which makes the algorithm robust to the outliers. This advantage is particularly significant in diagnostic/classification modeling in medicine and healthcare, where the number of samples is usually small, and outliers have a huge impact on the model training, leading to poor accuracy.

In a preliminary study [9], the GL algorithm was effective in classifying tasks with microarray gene expression data. In this manuscript we have significantly extended our preliminary work in the following ways:

1. providing a thorough description of the proposed GL algorithm as well as intuitive and qualitative explanations of scenarios where the new algorithm is superior to the Min-max and Z-score algorithms;
2. extending the GL algorithm to include a much better and more general parameter initialization for the non-convex optimization, which is a critical part of the algorithm for fitting the generalized logistic function to the empirical cumulative distribution function;
3. empirically demonstrating that the GL algorithm is not only effective in gene expression classification tasks, but also in a broad variety of different diagnostic/classification tasks with different types of variables.

### Data scaling formula

We model the values of a variable in the samples as a random variable (r.v.) *X*. In the GL algorithm, the scaled value *v*^′^ of a value, *v*, is obtained by

3 $$\begin{array}{*{20}l} v' = P_{X}(v), \end{array} $$

where *P*_*X*_(·) is the cumulative density function (CDF) of the r.v. *X*.

Using a CDF as a mapping can be also seen in the Histogram Equalization technique [10] in the field of Digital Image Processing for image contrast enhancement. The difference of the GL algorithm versus the Histogram Equalization technique is that we do not only use the CDF to scale the data, but also learn/approximate the functional expression of the CDF, so that it can be used to scale unseen values.

### Approximation of the cumulative density function

From the data, we do not know the exact functional form of the cumulative density function (CDF) of an variable whose value is represented by the r.v. *X*; therefore, we need to approximate the CDF. We can find the empirical cumulative density function (ECDF) using the formula

4 $$\begin{array}{*{20}l} \hat{P}_{X}(v) = \frac{1}{n}\sum_{i=1}^{n}1_{x_{i} \leq v}, \end{array} $$

where $\hat {P}_{X}(v)$ is the ECDF at a value *v*, *n* is the number of samples, and *x*_*i*_ is the value of the variable in the *i*^*th*^ sample.

Unfortunately, in most cases, the ECDF has no functional form expression. Moreover, the original data tend to be noisy, so the ECDF is usually very bumpy. Therefore, we use a generalized logistic (GL) function to approximate the ECDF. It has been proven that a logistic function can be used to accuractely approximate the CDF of a normal distribution [11]. In this algorithm, we do not make any assumption on the distribution of the data; therefore, we use a more general form of the logistic function, called the generalized logistic (GL) function

5 $$\begin{array}{*{20}l} L(x) = \frac{1}{\left(1+Qe^{-B(x-M)}\right)^{1/\nu}}.  \end{array} $$

Compared to the logistic function used in [11], this GL function provides the flexibility to approximate a more variety of distributions. One of the notable properties of (5) is that it maps the values in the interval (*∞*,−*∞*) to the interval (0,1). This property makes our GL algorithm robust to outliers, and guarantees that the scaled data will be in (0,1).

In order to approximate the ECDF, we need to learn the parameters *Q*, *B*, *M*, and *ν* from the data, so that the GL function could best fit the ECDF. The sum of squared differences of the GL function and the ECDF can be represented by

6 $$\begin{array}{*{20}l} \eta = \sum_{i=1}^{n}\left\|L(x_{i})-\hat{P}_{X}(x_{i})\right\|^{2}.  \end{array} $$

The best set of parameters is the minimizer of *η*, so the key to find the most appropriate GL function to approximate the ECDF is to solve an optimization problem

7 $$\begin{array}{*{20}l} \underset{B,M,Q,\nu}{\text{minimize}} \ \eta(B,M,Q,\nu). \end{array} $$

Because (5) and (6) are differentiable, the derivatives of *η* with respect to the parameters can be easily obtained, as shown in the following:

$$\begin{array}{*{20}l} \frac{d\eta}{dB} & = \sum_{i=1}^{n}-T_{1}\frac{Qe^{-B(x_{i}-M)}(x_{i}-M)}{T_{2}}, \\ \frac{d\eta}{dM} & = \sum_{i=1}^{n}T_{1}\frac{BQe^{-B(x_{i}-M)}}{T_{2}}, \\ \frac{d\eta}{dQ} & = \sum_{i=1}^{n}T_{1}\frac{e^{-B(x_{i}-M)}}{T_{2}}, \\ \frac{d\eta}{d \nu} & = \sum_{i=1}^{n}-T_{1}\frac{ln(Qe^{-B(x_{i}-M)}+1)}{\nu^{2}(Qe^{-B(x_{i}-M)}+1)^{1/\nu}}, \\ \text{where} \\ T_{1} & = 2(\hat{P}_{X}(x_{i})-L(x_{i})) \\ T_{2} & = \nu (Qe^{-B(x_{i}-M)}+1)^{1/\nu+1}. \end{array} $$

Therefore, a local minimum of (7) can be solved efficiently by any gradient descent optimization algorithms.

### Parameter initialization

The optimization problem described in (7) is non-convex, so in order to achieve a good local minimum (or even global minimum) of the objective function, the values of the parameters should be carefully initialized; i.e. determine *B*_0_,*M*_0_,*Q*_0_,*ν*_0_, which are the initialization of the parameters for the gradient descent iterations. By looking at the structure of the GL function, we can see that parameter *M* determines the “center” of the GL curve; therefore, parameter *M*, should be close to the median of the sample values. We first arrive at:

8 $$\begin{array}{*{20}l} M_{0} = \hat{P}_{X}^{-1}(0.5) = x_{med},  \end{array} $$

where *x*_*med*_ denotes the median value of the variable in the samples. From $L(x_{med}) \approx \hat {P}_{X}(x_{med}) \approx 0.5$, we have $L(x_{med}) = \frac {1}{(1+Q_{0}e^{-B_{0}(x_{med}-x_{med})})^{1/\nu _{0}}} \approx 0.5$, noting that we replace *M*_0_ by *x*_*med*_ because of (8). We obtain:

9 $$\begin{array}{*{20}l} \nu_{0} = \log_{2}(1+Q_{0}).  \end{array} $$

It is reasonable to assume that the minimum value in the samples will be scaled to a value close to 0.1, that is $L(x_{min}) \approx \hat {P}_{X}(x_{min}) \approx 0.1$, we have $L(x_{min}) = \frac {1}{(1+Q_{0}e^{-B_{0}(x_{min}-x_{med})})^{1/\nu _{0}}} \approx 0.1$, where *x*_*min*_ denotes the minimum value of the variable in the samples. We obtain:

10 $$\begin{array}{*{20}l} B_{0} = \frac{\ln\left((1+Q_{0})^{\log_{2}(10)}-1\right)-\ln(Q_{0})}{x_{med}-x_{min}}.  \end{array} $$

**Fig. 1:**
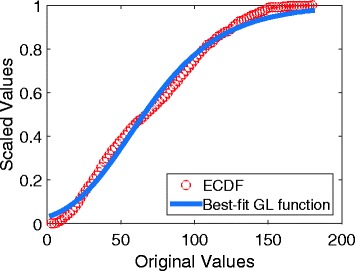
Fitting of the ECDF using the GL algorithm An example showing the approximation of an ECDF using a generalized logistic (GL) function

Now, *ν*_0_ and *B*_0_ are dependent on *Q*_0_. We further assume that the maximum value in the sample will be scaled to a value close to 0.9, that is $L(x_{max}) \approx \hat {P}_{X}(x_{max}) \approx 0.9$, thus $L(x_{max}) = \frac {1}{(1+Q_{0}e^{-B_{0}(x_{max}-x_{med})})^{1/\nu _{0}}} \approx 0.9$, where *x*_*max*_ denotes the maximum value of the variable in the samples. Combining (9) and (10), we obtain the following equation in terms of *Q*_0_:

11 $$\begin{array}{*{20}l} \frac{1}{1+Q_{0}e^{(\ln((1+Q_{0})^{\log_{2}(10)}-1)-\ln(Q_{0})) \frac{x_{max}-x_{med}}{x_{min}-x_{med}}}}=0.9^{\log_{2}(1+Q_{0})},  \end{array} $$

and the most suitable value for *Q*_0_ is the root of Eq. (11). The root can be resolved numerically and quickly by using the Newton’s method. With this initialization, we could find a set of parameters which make the GL function fit the ECDF well, as shown in Fig. 1.

### Qualitative comparisons of data scaling algorithms

In this section, we will intuitively and qualitatively discuss the scenarios where the GL algorithm is superior to the commonly used data scaling algorithms.

**Fig. 2:**
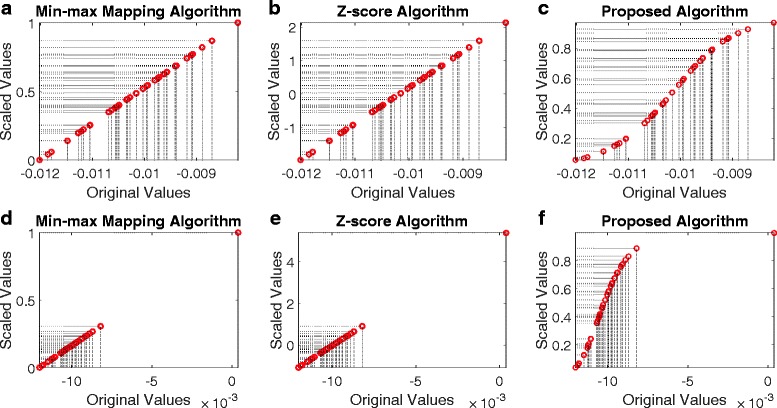
Behavior of data scaling algorithms with/without outliers. Top panels **a**–**c**: when there is no outlier in the data, the behavior of the Min-max algorithm, Z-score algorithm and the GL algorithm is very similar. Bottom panel **d**–**f**: when there is an outlier in the data, the behaviors of the Min-max algorithm and Z-score algorithm are significantly affected, but the impact of the outlier on the GL algorithm is neglectable

**The GL algorithm is robust to outliers** During the data collection period, the data might be corrupted for various reasons; e.g., system error, human error, sample contamination, etc. Therefore, a data de-noising or outlier detection procedure may be necessary in the data preprocessing step. The GL algorithm is intrinsically capable of handling situations where there are noisy samples and outliers in the samples. As Fig. 2a–c show, in the situation that there are no outliers in samples, all data scaling algorithms perform similarly. However, when an outlier exists in the data, as shown in Fig. 2d–e, the Min-max algorithm and the Z-score algorithm are affected by the outlier - the original values in the normal range are squeezed after the scaling. In contrast, the outlier’s impact to the GL algorithm is neglectable, as shown in Fig. 2f. Outliers are samples deviate strongly from the majority of (normal) samples, so the number of outliers will be always much smaller than the number of normal samples, and therefore, the contribution of outliers to the CDF of the samples is neglectable. However, outliers do not necessarily need to be the result of measurement errors, but may also occur due to variability, and represent completely valid instances. There are applications that are particularly concerned with such anomalies in the observations as they may carry valuable information about some rare modality of the processes responsible for its generation. For such applications, algorithms for outlier detection are utilized to interrogate the data and bring the focus to the rare signal in the data, and our data preprocessing algorithm is inappropriate to use for such purposes. Nevertheless, regardless of the outliers’ origin (error or variability), for the supervised task of classification, outliers are typically detrimental for classification accuracy, and their removal/correction is very welcome, if not necessary [12].

**Fig. 3:**
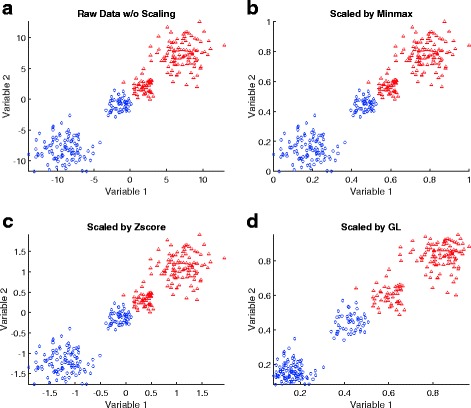
An 2D illustration on how the GL algorithm can affect the classification accuracy. **a** raw data without scaling; **b** data scaled by the Min-max algorithm; **c** data scaled by the Z-score algorithm; **d** data scaled by the GL algorithm

**The GL algorithm can improve classification accuracy** One of the complications which leads to poor classification accuracy is that the samples in different classes are dense and “crowded” near the decision boundary (otherwise, the accuracy would be expected to be high). Therefore, although in the training stage, the model can perfectly distinguish samples in different classes, in the testing stage, the model may make mistakes. Figure 3a shows an artificially generated data of two groups (red v.s. blue), and we can imagine those samples are used to test the classifier. Although the two groups of data are separable, a trained classifier may make mistakes because these data are not seen in the training. One way to improve the classification in the test is to enlarge the separation the data from two groups near the decision boundary. The intuition is that if the separation of two groups is by a large margin, it allows a wider variety of decision boundaries to separate the data. Because the Min-max algorithm and the Z-score algorithm are linear mappings, after the data are scaled, their relative distance will not change (Fig. 3b and c). In contrast, the GL algorithm is a nonlinear mapping; it will enlarge the distance of the dense samples that are located near the decision boundary, and squeeze the samples that are located away from the decision boundary (Fig. 3d). This effect reduces the classifier’s potential of making mistakes, thus improving the accuracy.

**Descriptions of datasets** We have included 16 datasets in our experiments. The tasks associated with the datasets cover a broad variety of diagnostic/classification problems in biomedical research. The information of the datasets, including the number of samples, variable types, and tasks, are summarized in Table 1. Among them, *LSVT*, *Pima Indian diabetes*, *Parkinsons*, *Wdbc*, *Breast tissue*, and *Indian liver* were downloaded from the UCI dataset repository (https://archive.ics.uci.edu/ml/datasets.html). These 6 datasets were selected because the majority of their variables are continuous, so that the data scaling algorithms could be applied (non-continuous variables were deleted). If a dataset was originally associated with a multiclass classification task, we will formulate a binary classification task as one-class-vs-others. The datasets, *Breast cancer*, *Colon cancer*, *Lung cancer*, *Prostate cancer*, and *Myeloma* were made available by Stantnikov et al. [13], and we downloaded the datasets from the supplementary material website (http://www.gems-system.org/). The datasets *DLBCL* and *Leukemia* were downloaded from the Kent Ridge Bio-medical Dataset Repository (http://datam.i2r.a-star.edu.sg/datasets/krbd); we removed the variables with missing values in the *DLBCL* dataset, so 715 variables were used in our experiments. The datasets *GSE 25869*, *GSE 27899IL*, and *GSE 29490*, were downloaded from the Gene Expression Omnibus Repository [14]. We converted the datasets to *.mat* format, and made them available to the public; please refer to Section “Availability of data and material” for details.

**Table 1:** Summary of datasets used in experiments (sorted by the no. of subjects in ascending order)

Dataset	No. of subjects (pos/neg)	Var. type	No. of var.	Task
*GSE 27899IL* [15]	10/10	DNA methylation	27578	diagnose ulcerative colitis
*Prostate cancer* [16]	14/9	microarray gene expression	15009	diagnose prostate cancer
*Colon cancer* [16]	15/11	microarray gene expression	15009	diagnose colon cancer
*Lung cancer* [16]	20/7	microarray gene expression	15009	diagnose lung cancer
*Breast cancer* [16]	17/15	microarray gene expression	15009	diagnose breast cancer
*Leukemia* [17]	11/27	microarray gene expression	7129	diagnose leukemia
*GSE 29490* [18]	20/7	DNA methylation	26916	diagnose colorectal carchinoma
*GSE 25869* [19]	14/9	DNA methylation	27570	diagnose gastric cancer
*Breast tissue* [20]	21/85	impedance measurements	9	diagnose breast tumor
*LSVT* [21]	42/84	wavelet and frequency based measurements	310	assessment of treatments in Parkinson
*DLBCL* [22]	88/72	microarray gene expression	715	diagnose DLBCL
*Myeloma* [23]	137/36	microarray gene expression	12625	diagnose bone lesions
*Parkinsons* [24]	147/48	vocal based measurements	22	diagnose Parkinson disease
*Wdbc* [25]	212/357	nuclear feature from image	30	diagnose breast tumor
*Indian liver* [26]	414/165	biochemistry based measurements	9	diagnose liver disease
*Pima Indians diabetes* [27]	268/500	clinical measurements	8	diagnose diabetes

**Evaluation methods** To assess how different data scaling algorithms affect classification performances, we used Logistic Regression (LR) and Support Vector Machine (SVM) as the classification models. These two classification models have been used extensively in biological and medical research due to their simplicity and accessibility. The program code of the experiments was implemented in MATLAB 8.4. The results were obtained using 5-fold cross-validations. One of the performance metrics we used was the area under the receiver operation characteristic curve (AUROC), which has been commonly used for binary classification performance evaluations; one of the advantages of AUROC is that its value does not depend on a classification score threshold. To have a more complete comparison of different data scaling methods and classification models, we also used accuracy (proportion of correct classifications). The threshold we used to determine the class labels (and thus, the accuracy) of the testing set samples was obtained by selecting a score which could maximize the accuracy in the training set; if multiple, or a range of scores could achieve the maximum accuracy, we would select the minimum. The mean value and 95 % confidence interval of the AUROC of each binary classification task can be found in Table 2, and the mean value and 95 % confidence interval of the proportion of correct classifications can be found in Table 3. Due to the large number of variables, the AUROC’s and accuracies of datasets *GSE 25869*, *GSE 27899IL*, and *GSE 29490* on Logistic Regression model were not available.

**Table 2:** Results of 16 datasets (16 binary classification tasks) using different data scaling algorithms and classification models

dataset	Method	None	Minmax	Zscore	GL
GSE27899IL	LR	NA ± NA	NA ± NA	NA ± NA	NA ± NA
	SVM	0.768 ± 0.104	0.814 ± 0.084	0.814 ± 0.074	**0.824 ± 0.058**
Prostate Cancer	LR	0.464 ± 0.000	0.749 ± 0.130	0.689 ± 0.156	**0.761 ± 0.108**
	SVM	0.573 ± 0.198	0.725 ± 0.232	0.713 ± 0.244	**0.822 ± 0.194**
Colon Cancer	LR	0.500 ± 0.000	0.895 ± 0.092	0.892 ± 0.082	**0.962 ± 0.046**
	SVM	0.670 ± 0.184	0.940 ± 0.058	0.937 ± 0.050	**0.981 ± 0.020**
Lung Cancer	LR	0.450 ± 0.000	0.839 ± 0.096	0.834 ± 0.108	**0.890 ± 0.050**
	SVM	0.397 ± 0.274	0.716 ± 0.136	0.710 ± 0.152	**0.774 ± 0.182**
Breast Cancer	LR	0.324 ± 0.000	0.809 ± 0.038	**0.821 ± 0.020**	0.819 ± 0.022
	SVM	0.708 ± 0.158	0.793 ± 0.052	0.795 ± 0.042	**0.812 ± 0.038**
Leukemia	LR	0.500 ± 0.000	0.988 ± 0.014	0.990 ± 0.006	**1.000 ± 0.000**
	SVM	0.935 ± 0.034	0.992 ± 0.010	0.991 ± 0.008	**1.000 ± 0.000**
GSE29490	LR	NA ± NA	NA ± NA	NA ± NA	NA ± NA
	SVM	0.983 ± 0.012	0.984 ± 0.034	0.985 ± 0.034	**0.994 ± 0.004**
GSE25869	LR	NA ± NA	NA ± NA	NA ± NA	NA ± NA
	SVM	0.935 ± 0.024	0.937 ± 0.020	0.938 ± 0.016	**0.943 ± 0.014**
Breast tissue	LR	0.520 ± 0.006	0.961 ± 0.032	**0.961 ± 0.044**	0.940 ± 0.054
	SVM	0.713 ± 0.108	0.968 ± 0.006	0.970 ± 0.014	**0.972 ± 0.010**
LSVT	LR	0.500 ± 0.000	0.875 ± 0.008	0.846 ± 0.022	**0.921 ± 0.012**
	SVM	0.500 ± 0.000	0.879 ± 0.012	0.863 ± 0.014	**0.919 ± 0.020**
DLBCL	LR	0.601 ± 0.038	0.608 ± 0.038	0.610 ± 0.048	**0.660 ± 0.062**
	SVM	0.616 ± 0.050	0.622 ± 0.052	0.619 ± 0.052	**0.654 ± 0.054**
Myeloma	LR	0.500 ± 0.000	0.729 ± 0.044	0.739 ± 0.072	**0.746 ± 0.038**
	SVM	0.573 ± 0.098	0.748 ± 0.052	0.747 ± 0.054	**0.750 ± 0.054**
Parkinsons	LR	0.875 ± 0.012	0.896 ± 0.054	0.893 ± 0.058	**0.906 ± 0.048**
	SVM	0.882 ± 0.010	0.875 ± 0.010	0.885 ± 0.024	**0.891 ± 0.018**
Wdbc	LR	0.942 ± 0.002	0.982 ± 0.004	0.978 ± 0.006	**0.993 ± 0.004**
	SVM	0.990 ± 0.002	0.994 ± 0.002	0.993 ± 0.004	**0.995 ± 0.000**
Indian Liver	LR	0.680 ± 0.002	0.743 ± 0.008	0.742 ± 0.008	**0.746 ± 0.010**
	SVM	0.636 ± 0.068	0.696 ± 0.008	0.692 ± 0.034	**0.695 ± 0.008**
Pima Indians Diabetes	LR	0.604 ± 0.004	0.827 ± 0.004	0.827 ± 0.004	**0.834 ± 0.006**
	SVM	0.826 ± 0.004	0.828 ± 0.006	0.828 ± 0.006	**0.834 ± 0.006**

The performances are measured by the average Area Under the ROC in 5-fold cross-validations. The means and 95 % confidence intervals are included. Column names: None - no data scaling; Minmax - Min-max algorithm; Z-score - Z-score algorithm; GL - GL algorithm. Best performances are emphasized in bold

**Table 3:** Results of 16 datasets (16 binary classification tasks) using different data scaling algorithms and classification models

dataset	Method	None	Minmax	Zscore	GL
GSE27899IL	LR	NA ± NA	NA ± NA	NA ± NA	NA ± NA
	SVM	0.770 ± 0.054	**0.780 ± 0.134**	**0.780 ± 0.134**	**0.780 ± 0.134**
Prostate Cancer	LR	0.609 ± 0.000	0.757 ± 0.132	0.722 ± 0.078	**0.765 ± 0.100**
	SVM	0.635 ± 0.078	0.748 ± 0.156	0.748 ± 0.156	**0.835 ± 0.114**
Colon Cancer	LR	0.577 ± 0.000	0.877 ± 0.064	0.877 ± 0.064	**0.923 ± 0.054**
	SVM	0.677 ± 0.178	0.900 ± 0.042	0.915 ± 0.034	**0.946 ± 0.042**
Lung Cancer	LR	0.741 ± 0.000	0.859 ± 0.062	0.852 ± 0.052	**0.896 ± 0.062**
	SVM	0.778 ± 0.052	0.859 ± 0.034	0.859 ± 0.034	**0.867 ± 0.040**
Breast Cancer	LR	0.773 ± 0.000	0.918 ± 0.040	**0.955 ± 0.000**	**0.955 ± 0.000**
	SVM	0.827 ± 0.040	0.909 ± 0.000	0.909 ± 0.000	**0.936 ± 0.050**
Leukemia	LR	0.710 ± 0.000	0.956 ± 0.030	0.965 ± 0.030	**1.000 ± 0.000**
	SVM	0.939 ± 0.030	0.965 ± 0.030	0.965 ± 0.030	**1.000 ± 0.000**
GSE29490	LR	NA ± NA	NA ± NA	NA ± NA	NA ± NA
	SVM	0.942 ± 0.034	0.954 ± 0.034	0.958 ± 0.034	**0.979 ± 0.000**
GSE25869	LR	NA ± NA	NA ± NA	NA ± NA	NA ± NA
	SVM	0.891 ± 0.038	0.891 ± 0.044	0.894 ± 0.034	**0.897 ± 0.034**
Breast tissue	LR	0.778 ± 0.016	0.930 ± 0.010	**0.930 ± 0.016**	0.927 ± 0.016
	SVM	0.681 ± 0.220	0.932 ± 0.024	0.926 ± 0.020	**0.942 ± 0.008**
LSVT	LR	0.500 ± 0.000	0.870 ± 0.012	0.824 ± 0.038	**0.915 ± 0.002**
	SVM	0.500 ± 0.000	0.873 ± 0.036	0.858 ± 0.036	**0.908 ± 0.006**
DLBCL	LR	0.567 ± 0.014	0.571 ± 0.014	0.579 ± 0.032	**0.602 ± 0.074**
	SVM	0.594 ± 0.082	0.592 ± 0.064	0.585 ± 0.044	**0.600 ± 0.100**
Myeloma	LR	0.792 ± 0.000	0.805 ± 0.020	0.804 ± 0.018	**0.805 ± 0.026**
	SVM	0.794 ± 0.006	0.809 ± 0.014	0.807 ± 0.026	**0.813 ± 0.020**
Parkinsons	LR	0.865 ± 0.006	**0.894 ± 0.022**	0.891 ± 0.016	0.868 ± 0.006
	SVM	0.880 ± 0.016	**0.884 ± 0.006**	0.877 ± 0.020	0.868 ± 0.016
Wdbc	LR	0.878 ± 0.002	0.965 ± 0.010	0.963 ± 0.012	**0.971 ± 0.012**
	SVM	0.960 ± 0.010	0.979 ± 0.004	0.976 ± 0.002	**0.980 ± 0.008**
Indian Liver	LR	0.716 ± 0.002	0.727 ± 0.014	0.733 ± 0.008	**0.736 ± 0.006**
	SVM	0.719 ± 0.006	0.720 ± 0.014	0.718 ± 0.010	**0.720 ± 0.008**
Pima Indians Diabetes	LR	0.490 ± 0.070	0.738 ± 0.010	0.738 ± 0.010	**0.740 ± 0.012**
	SVM	0.734 ± 0.052	**0.765 ± 0.008**	0.753 ± 0.040	0.748 ± 0.034

The performances are measured by the average proportion of correct classification in 5-fold cross-validations. The means and 95 % confidence intervals are included. Column names: None - no data scaling; Minmax - Min-max algorithm; Zscore - Z-score algorithm; GL - GL algorithm. Best performances are emphasized in bold

## Results and discussions

In most of the classification tasks, models learned with unscaled data have the worst performances. This is consistent with our expectations. In general, an appropriate data processing step (i.e., data scaling) is able to improve the accuracy of a model. Comparing the GL algorithm to the Z-score algorithm and the Min-max algorithm, in most tasks, models learned with the data scaled by the GL algorithm achieved the best average AUROC’s and the best average accuracies. Specifically, in the experiments, out of the 29 task-model cases (16 tasks; 2 models per task, but LR was not available in 3 tasks), the GL algorithm achieve the best AUROC’s in 27 cases and the best accuracies in 25 cases. The advantage of the GL algorithm was more notable in datasets with a small number of samples, such as *colon*, *lung*, and *prostate*, in which the existence of outliers may significantly affects the model performance. For example, in the colon cancer diagnostic task, while using the SVM classifier, the model learned using GL scaled data achieved a 0.822 AUROC, while the best AUROC achieved by the SVM classifier from other data scaling methods was 0.725; it was a 13.4 % of improvement. The improvements of AUROC using the data scaled by the GL algorithm were less notable in the tasks *Parkinsons*, *Wdbc*, *Indian liver*, and *Pima Indians Diabetes*. One of the reasons was that the number of samples in those data sets was relatively large, so the negative effects of outliers became less significant; another possible reason was that before the contributors uploaded the data set, they might have performed a preprocessing step to correct/remove abnormal samples. It is worthwhile to point out that, in three task-model cases (i.e., RL and SVM in the *Parkinsons* task, and SVM in the *Pima Indians Diabetes* task), although the GL algorithm achieved the best AUROC’s, it did not achieve the best accuracies. That might be due to the the threshold selection rule in our experiments; while the AUROC’s of different task-model cases were close, the ranking of the accuracies would be very sensitive to the selected threshold.

## Conclusion

In this article, we present a simple yet effective data scaling algorithm, the GL algorithm, to scale data to an appropriate interval for diagnostic and classification modeling. In the GL algorithm, the values of a variable are scaled in the (0,1) interval using the cumulative density function of the variable. Since obtaining the functional expression of the CDF is difficult, a generalized logistic GL function is used to fit the empirical cumulative distribution function, and the optimized GL function is used for data scaling. The GL algorithm is intrinsically robust to outliers, so it is particularly suitable for diagnostic/classification models in clinical/medical applications, where the number of samples is usually small; it scales the data in a nonlinear fashion, which leads to improvement of accuracy. Experimental results show that models learned using data scaled by the GL algorithm generally outperform the ones using the Min-max algorithm and the Z-score algorithm, which are currently the most commonly used data scaling algorithms.

## Abbreviations

AUROC: Area under the receiver operating characteristic curve
CDF: Cumulative density function
ECDF: Empirical cumulative density function
GL: Generalized logistic
LR: Logistic regression
ROC: Receiver operating characteristic
SVM: Support vector machine

## References

[1] Kourou K, Exarchos TP, Exarchos KP, Karamouzis MV, Fotiadis DI (2015). Machine learning applications in cancer prognosis and prediction. Comput Struct Biotechnol J.

[2] Swan AL, Mobasheri A, Allaway D, Liddell S, Bacardit J (2013). Application of machine learning to proteomics data: classification and biomarker identification in postgenomics biology. Omics: J Integr Biol.

[3] Kelchtermans P, Bittremieux W, Grave K, Degroeve S, Ramon J, Laukens K, Valkenborg D, Barsnes H, Martens L (2014). Machine learning applications in proteomics research: How the past can boost the future. Proteomics.

[4] Foster KR, Koprowski R, Skufca JD (2014). Machine learning, medical diagnosis, and biomedical engineering research-commentary. Biomed Eng Online.

[5] Maltoni M, Caraceni A, Brunelli C, Broeckaert B, Christakis N, Eychmueller S, Glare P, Nabal M, Vigano A, Larkin P (2005). Prognostic factors in advanced cancer patients: evidence-based clinical recommendations–a study by the steering committee of the european association for palliative care. Journal of Clinical Oncology.

[6] Han J, Kamber M, Pei J (2011). Data Mining: Concepts and Techniques: Concepts and Techniques.

[7] Haykin SS (2009). Neural Networks and Learning Machines.

[8] Dudoit S, Yang YH, Callow MJ, Speed TP (2002). Statistical methods for identifying differentially expressed genes in replicated cdna microarray experiments. Stat Sin..

[9] Cao XH, Obradovic Z (2015). A robust data scaling algorithm for gene expression classification. Bioinformatics and Bioengineering (BIBE), 2015 IEEE 15th International Conference On.

[10] Gonzalez R, Woods R (2008). Digital image processing.

[11] Bowling SR, Khasawneh MT, Kaewkuekool S, Cho BR (2009). A logistic approximation to the cumulative normal distribution. J Ind Eng Manag.

[12] Acuna E, Rodriguez C. A meta analysis study of outlier detection methods in classification. Technical paper, Department of Mathematics, University of Puerto Rico at Mayaguez. 2004.

[13] Statnikov A, Tsamardinos I, Dosbayev Y, Aliferis CF (2005). Gems: a system for automated cancer diagnosis and biomarker discovery from microarray gene expression data. Int J Med Inform.

[14] Edgar R, Domrachev M, Lash AE (2002). Gene expression omnibus: Ncbi gene expression and hybridization array data repository. Nucleic Acids Res.

[15] Häsler R, Feng Z, Bäckdahl L, Spehlmann ME, Franke A, Teschendorff A, Rakyan VK, Down TA, Wilson GA, Feber A (2012). A functional methylome map of ulcerative colitis. Genome Res.

[16] Ramaswamy S, Tamayo P, Rifkin R, Mukherjee S, Yeang CH, Angelo M, Ladd C, Reich M, Latulippe E, Mesirov JP (2001). Multiclass cancer diagnosis using tumor gene expression signatures. Proc Natl Acad Sci.

[17] Golub TR, Slonim DK, Tamayo P, Huard C, Gaasenbeek M, Mesirov JP, Coller H, Loh ML, Downing JR, Caligiuri MA (1999). Molecular classification of cancer: class discovery and class prediction by gene expression monitoring. Science.

[18] Kibriya MG, Raza M, Jasmine F, Roy S, Paul-Brutus R, Rahaman R, Dodsworth C, Rakibuz-Zaman M, Kamal M, Ahsan H (2011). A genome-wide dna methylation study in colorectal carcinoma. BMC Med Genet.

[19] Kwon OH, Park JL, Kim M, Kim JH, Lee HC, Kim HJ, Noh SM, Song KS, Yoo HS, Paik SG (2011). Aberrant up-regulation of lamb3 and lamc2 by promoter demethylation in gastric cancer. Biochem Biophys Res Commun.

[20] Jossinet J (1996). Variability of impedivity in normal and pathological breast tissue. Med Biol Eng Comput.

[21] Tsanas A, Little MA, Fox C, Ramig LO (2014). Objective automatic assessment of rehabilitative speech treatment in parkinson’s disease. IEEE Trans Neural Syst Rehabil Eng.

[22] Rosenwald A, Wright G, Chan WC, Connors JM, Campo E, Fisher RI, Gascoyne RD, Muller-Hermelink HK, Smeland EB, Giltnane JM (2002). The use of molecular profiling to predict survival after chemotherapy for diffuse large-b-cell lymphoma. N Engl J Med.

[23] Tian E, Zhan F, Walker R, Rasmussen E, Ma Y, Barlogie B, Shaughnessy Jr JD (2003). The role of the wnt-signaling antagonist dkk1 in the development of osteolytic lesions in multiple myeloma. N Engl J Med.

[24] Little MA, McSharry PE, Hunter EJ, Spielman J, Ramig LO (2009). Suitability of dysphonia measurements for telemonitoring of parkinson’s disease. IEEE Trans Biomed Eng.

[25] Street WN, Wolberg WH, Mangasarian OL (1993). Nuclear feature extraction for breast tumor diagnosis. IS&T/SPIE’s Symposium on Electronic Imaging: Science and Technology.

[26] Ramana BV, Babu MSP, Venkateswarlu N (2011). A critical study of selected classification algorithms for liver disease diagnosis. Int J Database Manag Syst.

[27] Smith JW, Everhart J, Dickson W, Knowler W, Johannes R. Using the adap learning algorithm to forecast the onset of diabetes mellitus. In: Proceedings of the Annual Symposium on Computer Application in Medical Care. American Medical Informatics Association: 1988. p. 261.

